# Chlorine inhalation-induced myocardial depression and failure

**DOI:** 10.14814/phy2.12439

**Published:** 2015-06-24

**Authors:** Ahmed Zaky, Wayne E Bradley, Ahmed Lazrak, Iram Zafar, Stephen Doran, Aftab Ahmad, Carl W White, Louis J Dell'Italia, Sadis Matalon, Shama Ahmad

**Affiliations:** 1 Department of Anesthesiology and Perioperative Medicine, University of Alabama at BirminghamBirmingham, Alabama; 2 Department of Medicine, Birmingham Veteran Affairs Medical CenterBirmingham, Alabama; 3 Division of Cardiovascular Disease, University of Alabama Medical CenterBirmingham, Alabama; 4 Department of Pediatrics, University of Colorado DenverBoulder, Colorado

**Keywords:** Coronary sinus, echocardiography, halogen, left ventricular dysfunction

## Abstract

Victims of chlorine (Cl_2_) inhalation that die demonstrate significant cardiac pathology. However, a gap exists in the understanding of Cl_2_-induced cardiac dysfunction. This study was performed to characterize cardiac dysfunction occurring after Cl_2_ exposure in rats at concentrations mimicking accidental human exposures (in the range of 500 or 600 ppm for 30 min). Inhalation of 500 ppm Cl_2_ for 30 min resulted in increased lactate in the coronary sinus of the rats suggesting an increase in anaerobic metabolism by the heart. There was also an attenuation of myocardial contractile force in an ex vivo (Langendorff technique) retrograde perfused heart preparation. After 20 h of return to room air, Cl_2_ exposure at 500 ppm was associated with a reduction in systolic and diastolic blood pressure as well echocardiographic/Doppler evidence of significant left ventricular systolic and diastolic dysfunction. Cl_2_ exposure at 600 ppm (30 min) was associated with biventricular failure (observed at 2 h after exposure) and death. Cardiac mechanical dysfunction persisted despite increasing the inspired oxygen fraction concentration in Cl_2_-exposed rats (500 ppm) to ameliorate hypoxia that occurs after Cl_2_ inhalation. Similarly ex vivo cardiac mechanical dysfunction was reproduced by sole exposure to chloramine (a potential circulating Cl_2_ reactant product). These results suggest an independent and distinctive role of Cl_2_ (and its reactants) in inducing cardiac toxicity and potentially contributing to mortality.

## Introduction

Exposure to chlorine (Cl_2_) gas remains an ongoing health concern, both via its possible use in chemical warfare and accidental exposure during industrial manufacturing and transport. Toxicity of Cl_2_ is a complex phenomenon. It consists of a primary injury to the airways and alveolar epithelia and subsequent escalation of damage by more stable secondary reactants (Bessac and Jordt 2010; Samal et al. 2010; White and Martin 2010; Yadav et al. 2010; Zarogiannis et al. 2014; Mo et al. 2015). Concentration of Cl_2_, duration of exposure and susceptibility of the individual contribute significantly to the biological response (Barrow et al. 1979; Squadrito et al. 2010; Mo et al. 2013). Cl_2_ inhalation results in profound respiratory and cardiovascular morbidity (Bessac and Jordt 2010; Samal et al. 2010; White and Martin 2010; Yadav et al. 2010). The range of clinical findings in persons exposed to high levels of Cl_2_ include; asphyxia with respiratory failure, pulmonary edema, acute pulmonary hypertension, cardiomegaly, pulmonary vascular congestion, acute burns of upper and proximal lower airways, airway hyper-responsiveness to methacholine (White and Martin 2010; Song et al. 2011; Fanucchi et al. 2012; Balte et al. 2013; Gessner et al. 2013). Whereas Cl_2-_induced pulmonary effects are witnessed on scene, cardiac effects of Cl_2_ are described primarily in human autopsy reports (Suzuki et al. 2001; Wenck et al. 2007; Kose et al. 2009; White and Martin 2010). A severely dilated right heart was observed in victims (that died) of World War I Cl_2_ poisoning (Arthur Hurst 1917). It is not clear, however, whether cardiac dilatation resulted from a primary and distinctive cardiac dysfunction or from pulmonary dysfunction. Cardiac dysfunction resulting from inhalational Cl_2_ exposure could result from pulmonary hypertension due to severe lung injury and/or hypoxemia, or from a distinct insult to the heart mediated either by the release of vasoactive mediators such as endothelin, and/or by the reaction of Cl_2_, or its metabolites with important signaling mediators (e.g., NO). Cardiovascular dysfunction can also be caused and aggravated by inhalation of oxidant gases, environmentally persistent free radicals potentially derived from combustion of Cl_2_-containing hydrocarbons and other environmental pollutants (Pham et al. 2009; Lord et al. 2011; Rappold et al. 2011; Devlin et al. 2012). Multiple human and animal studies have demonstrated a picture of right ventricular failure resulting from Cl_2_- induced increase in pulmonary vascular resistance (Winternitz et al. 1920; Gunnarsson et al. 1998; Wang et al. 2002; Wang et al. 2004). Cl_2_ inhalation was also shown to cause injury to both pulmonary and systemic vasculatures (Samal et al. 2010; Honavar et al. 2011, 2014).

Exposure to 600 ppm Cl_2_ causes greater than 90% mortality within 24 h in mouse models (Zarogiannis et al. 2011). We previously demonstrated decreased heart rate, loss of cardiac ATP contents, and apoptotic cell death in rats inhaling Cl_2_ (500 ppm for 30 min) (Ahmad et al. 2014, 2015). This was potentially attributed to the formation of circulating Cl_2_ reactants and chlorination and inactivation of cardiac sarcoendoplasmic Ca^2+^ ATPase (SERCA) causing cytosolic Ca^2+^ overload. The objectives of this study were to (1) characterize the effects of Cl_2_ on cardiac function at two different levels of exposure; and (2) explore whether Cl_2_-induced cardiotoxicity is dependent on hypoxia. We demonstrate that inhalation of Cl_2_ (500 or 600 ppm for 30 min) results in severe cardiac injury (assessed by echochardiography in vivo and force of contraction measurements in isolated perfused hearts) which persists even when arterial hypoxemia (resulting from lung injury) is alleviated by the inhalation of supplemental oxygen.

## Materials and Methods

### Rat exposure to chlorine gas

All procedures were approved by the University of Alabama at Birmingham Institutional Animal Care and Use Committee. Whole body Cl_2_ exposure of male Sprague–Dawley rats (200–250 g, Harlan, Indianapolis, IN) was performed as described previously (Leustik et al. 2008; Ahmad et al. 2015). Briefly, rats were exposed to 500 ppm or 600 ppm chlorine for 30 min. Rats were then returned to room air and monitored continuously up to 6 h thereafter at 20 h. Pulse oximetry was performed using a MouseOx (Starr Life Sciences, Oakmont, PA) system. In a group of animals, normal oxygen saturations (as measured by pulse oximetry) were restored by housing rats in 40% oxygen containing chambers where they were continuously monitored up to 20 h after Cl_2_ exposure.

### Blood gas measurements

Blood from the descending aorta was collected as described before (Ahmad et al. 2015). Sampling from Coronary sinus (CS) was performed under mechanical ventilation using 95% oxygen and anesthesia. The blood was injected into a precalibrated test card (EPOC-BGEM Test Card) of an EPOC Blood gas analysis system (Heska, Loveland, CO).

### Small animal hemodynamics

All experiments were performed under 2% isoflurane anesthesia. The body temperature was maintained at 37°C during measurements. For dP/dt measurements, a 2 F high-fidelity micromanometer catheter (SPR-671, Millar Institute, Houston, TX) was inserted into the right carotid artery and advanced to the LV. Using a Biopac MP100 system with AcqKnowledge 3.7.3 software (BIOPAC, Goleta, CA), left ventricular end diastolic pressure (LVEDP), left ventricular peak systolic pressure (LVPSP), and the peak rate of rise of ventricular pressure at systole (dP/dt maximum) and its reduction at diastole (dP/dt minimum) were measured. Systemic blood pressure was also monitored with a Millar pressure catheter inserted in the femoral artery.

### Echocardiography

Cardiac images were acquired in anesthetized animals (ketamine 80 mg/kg) using a Vevo2100 high-resolution ultrasound system (VisualSonics Inc., Toronto, ON) with a 13-MHz to 24-MHz linear transducer (MS-250). Rats were placed supine on the warmed stage of the echocardiography system. Parasternal long- and short-axis two-chamber M-mode and B-mode views were obtained at mid papillary level and averaged to determine LV dimensions at end-systole and end-diastole. LV volumes, cardiac output, fractional shortening, and ejection fraction were obtained. Apical 4-chamber B- and M-modes were obtained to determine tricuspid annular plane systolic excursion (TAPSE), transmitral flow and septal mitral annular tissue displacement. Spectral Doppler was used to determine transmitral early (*E*) and atrial (*A*) wave peak velocities, isovolumic relaxation time (IVRT), *E*-wave deceleration time, and isovolumic contraction time, with the ratio of *E* to *A* calculated. Peak early (*E′*), atrial (*A*′), and systolic (s=) annular velocities were recorded, and (*E)* to *(E′)* ratios were calculated. LV fractional shortening (LVFS), LV ejection fraction (LVEF), left ventricular end-systolic volume (LVESV) and end diastolic volume (LVESD), and end-systolic and end diastolic dimensions were measured for the assessment of LV systolic function. The 2D echocardiographic images acquired from the parasternal long-axis and short-axis views were analyzed using 2D speckle-tracking software (VevoStrain Analysis; VisualSonics Inc. Toronto, ON). Each image of the LV myocardium was divided into six standard anatomic segments throughout the cardiac cycle. Longitudinal and transverse strains were assessed from long-axis views, whereas radial and circumferential strains were assessed from short-axis views. A (−) sign was given for a decrease in dimension and a (+) sign was given for an increase in dimension. We measured peak systolic global strain. All strain data were measured over three heartbeats and averaged. Two observers with expertise in echocardiography assessed the studies for intra- and interobserver reproducibility of longitudinal, transverse circumferential, and radial tracking.

Isolated heart preparation: Isolated hearts were retrogradely perfused using modified Langendorff technique as described before (McNicholas-Bevensee et al. 2006; Bell et al. 2011). Briefly, rats were anesthetized using 40 mg/kg pentobarbital (i.p). Chest was opened and the heart quickly excised and placed in Tyrode's buffer. The heart was cannulated via the aorta and perfused (using Tyrode's buffer) on a modified Lagendorff apparatus. A string was tied to the heart as described before (Bell et al. 2011) and its other end was attached to a microtransducer to record the force of contraction.

### Statistical analysis

Means were compared by two-tailed *t*-test for comparison between two groups and one-way analysis of variance (ANOVA) for multiple comparisons. All echocardiography analysis and calculations were performed using SPSS version 19.0 (SPSS Inc., Chicago, IL).

## Results

**Figure 1:**
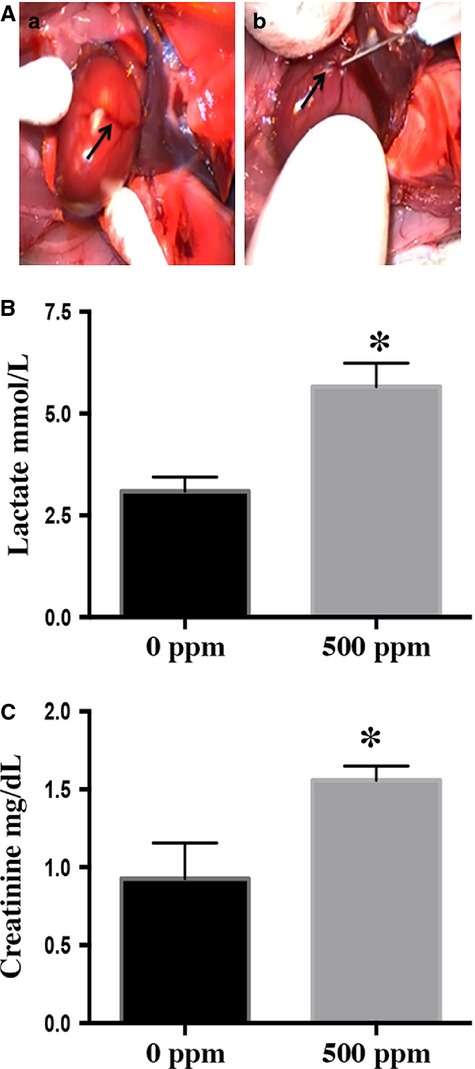
Chlorine exposure causes myocardial injury. Rats were exposed to 500 ppm Cl_2_ for 30 min and transferred to room air. (A) Blood was collected from the coronary sinus (CS, here black arrow in ‘a’ points the CS and in ‘b’ demonstrates the blood draw) for analysis of lactate and creatinine (B & C). Values shown are mean ± SEM (*n* = 3). *indicates significant (*P* < 0.05) difference from the 0 ppm controls.

We observed significant decrease in heart rates (451 ± 8 bpm at 0 ppm vs. 322 ± 10 bpm at 500 ppm) and decreased oxygen saturations (96 ± 1% at 0 ppm vs. 86 ± 2% at 500 ppm) when rats were exposed to 500 ppm Cl_2_ (30 min) and returned to room air for up to 20 h. There was an increase in arterial blood (AB) and coronary sinus (CS) lactate of Cl_2_-inhaling rats (1.38 ± 0.05 mg/dL at 0 ppm vs. 5.33 ± 1.93 mg/dL at 500 ppm in AB when measured at 3 h after Cl_2_ exposure). Coronary sinus (CS) lactate levels were elevated in rats exposed to Cl_2_ (500 ppm) that were then returned to room air for 20 h (Fig.1A and B). Interestingly, CS creatinine was also elevated in Cl_2_-inhaling rats (500 ppm) (Fig.1C).

**Figure 2:**
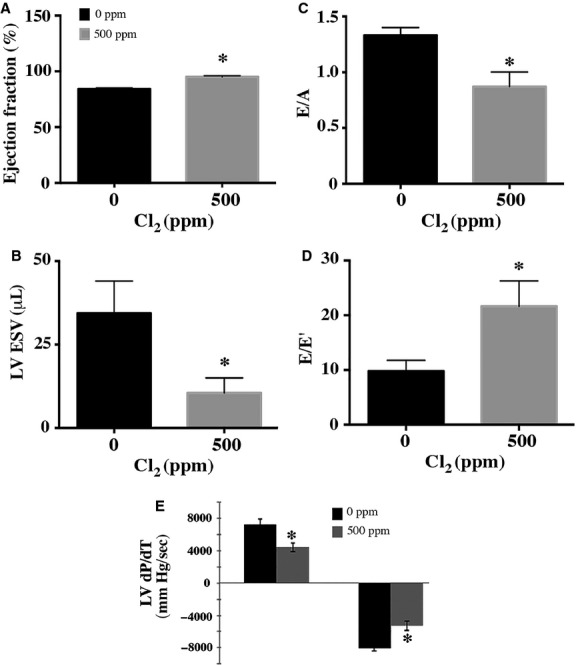
Chlorine exposure causes cardiac dysfunction. Rats were exposed to 500 ppm Cl_2_ for 30 min and transferred to room air. Twenty hours later echocardiography was performed as described in the Methods section. (A) A significant increase in ejection fraction (EF), (B) Significant reduction in LV ESV values explaining the increase in LV EF, (C) A significant reduction in the ratio of early to late transmitral diastolic velocities *E*/*A* denoting diastolic dysfunction, (D) A significant increase in the ratio of early diastolic transmitral (*E*) to early diastolic mitral annular velocity (*E*)′, (*E*/*E*′), another sign of diastolic dysfunction, E shows LV dP/dT maximum and minimum in controls and rats exposed to chlorine at 500 ppm. On the left there is a significant decline of LV dP/dt maximum compared to controls denoting global contractile dysfunction. On the right there is a significant reduction in LV dP/dt minimum compared to controls denoting a relaxation abnormality and diastolic dysfunction. dP/dt maximum; rate of rise of ventricular pressure in early systole, dP/dt minimum; rate of decline of ventricular pressure in early diastole. Results are representative of three independent experiments. Values shown are mean ± SEM (*n* = 6). *indicates significant (*P* < 0.05) difference from the 0 ppm controls. LV, left ventricle; (E), early diastolic transmitral peak wave velocity, (A); late atrial diastolic wave peak velocity; LV ESV, left ventricular end-systolic volume.

The in vivo cardiac effects of Cl_2_ were explored by surface echocardiography. Echocardiography was performed at 20 h after Cl_2_ inhalation under ketamine anesthesia. The results showed a significant decrease in left ventricular end-systolic diameter (LVESD) and volume (LVESV). LVEDV was unchanged, resulting in a significant increase in LV ejection fraction (LVEF) (Fig.2A and B) in the face of a decrease in both aortic diastolic and systolic BPs (80.6781 ± 22.028 mmHg for 0 ppm vs. 63 ± 4 mmHg diastolic BP for 500 ppm; 135 ± 2 mmHg for 0 ppm vs. 104.7 ± 4.66 mmHg systolic BP for 500 ppm). However, despite the increased LV shortening and decreased arterial pressure, there was a significant decrease in diastolic function manifested by a significant decrease in the ratio of early (*E*) to late (*A*) peak diastolic velocities across the mitral valve (*E*/*A*) and a significant reduction in early diastolic mitral annular tissue velocity (*E*′) as well as a significant increase in the ratio of early diastolic transmitral peak velocity to early mitral annular tissue velocity (*E*/*E*′) (Fig.2C and D).

There was a reduction in both (dP/dt maximum) (4425 ± 542 at 500 ppm Cl_2_ vs. 7270 ± 62, *P* = 0.027) at 0 ppm Cl_2_) and (dP/dt minimum) (5301 ± 572 at 500 ppm Cl_2_ vs. 8028 ± 369, *P* = 0.016 at 0 ppm Cl_2_) (Fig.2E). A direct myocardial depressant effect of Cl_2_ was also supported by the studies of the ex vivo Langendorff retrograde perfused heart preparation in rats exposed to Cl_2_ and returned to room air for 20 h. Cardiac force of contraction was significantly reduced 20 h after Cl_2_ exposure (14.53 ± 4.18 mN in 0 ppm vs. 4.80 ± 1.80 mN in 500 ppm). The heart/body weight ratio of Cl_2_-exposed animals was significantly increased (0.003667 ± 0.00006 for 0 ppm vs. 0.0044 ± 0.00005 for 500 ppm at 20 h postexposure duration), reflecting an increase in extravascular cardiac fluid.

**Figure 3:**
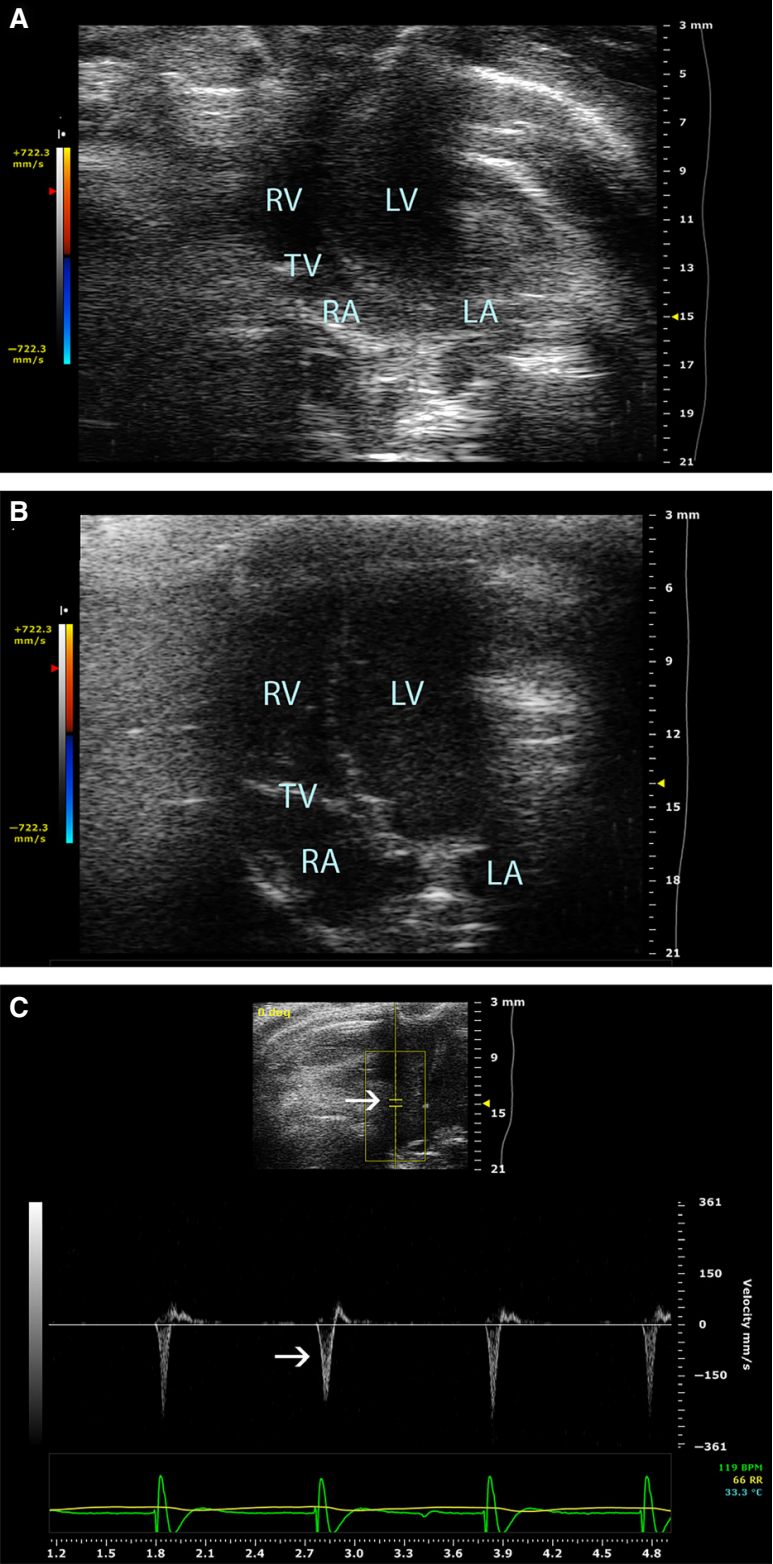
Apical four-chamber view of controls and Cl_2_-exposed rats at 600 ppm. (A) 2-D image of apical four-chamber view of controls with normal chamber dimensions. (B) 2-D image of an apical four-chamber view of Cl_2_-exposed rats at 600 ppm showing biventricular dilatation observed after 2 h of exposure. C. Spectral doppler representation of an apical four-chamber view showing acute severe tricuspid regurgitation upon 600 ppm Cl_2_ inhalation (arrows indicate measurments of tricuspid valve regurgitation velocities). RV (right ventricle); LV (left ventricle); TV (tricuspid valve); RA (right atrium); LA (left atrium); BPM (beats/min); RR (respiratory rate).

Human exposures could be more severe as concentrations of Cl_2_ for a 30 min period encountered during the Graniteville train derailment were 6868, 837, and 59 ppm at 0.2, 0.5, and 1 km downwind from the epicenter of the accident (Buckley et al. 2012; Balte et al. 2013). Therefore, to further investigate clinically relevant contribution of cardiac toxicity to Cl_2_-induced mortality, we exposed rats to a higher dose, 600 ppm Cl_2_, for 30 min and performed echocardiography at 2 h postexposure, an approximate time when the animals start to succumb. A similar dose of chlorine given for 45 min would result in about 90% fatality within 24 h postexposure in mice (Zarogiannis et al. 2011). Echocardiographic examination of this group showed severe biventricular systolic dysfunction manifested as a drastic decline in LVEF, fractional area shortening (FAC), and new-onset of acute severe tricuspid regurgitation which resulted from acute right ventricular annular dilatation (Fig.3). Most of these rats died during echocardiography.

**Figure 4:**
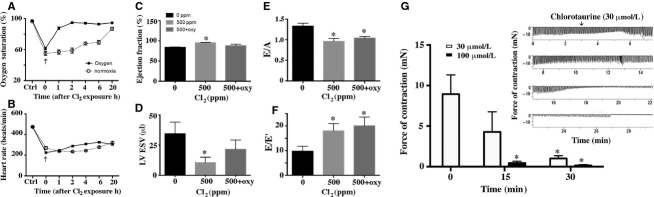
Effect of oxygen supplementation on Cl_2_-induced cardiotoxicity. Rats were exposed to Cl_2_ (500 ppm, 30 min), their pulse ox was measured and then they were placed in 40% oxygen containing environment from where they were removed one at a time to perform the pulse ox (part A & B). After 20 h echocardiography was performed as described in the legend to Fig.2. Ex vivo force of contraction was observed in retrograde perfused rat hearts with or without freshly prepared taurochloramine (part G). Inset of part G shows a profile of force of contraction recorded during the duration of experiment). Values shown are mean ± SEM (*n* = 4). *indicates significant (*P* < 0.05) difference from the 0 ppm controls.

The airways and lungs are the first targets of Cl_2_ that results in dyspnea and decreased oxygen saturations. We demonstrated decreased oxygen saturations and occurrence of hypoxia in the hearts of rats exposed to Cl_2_ (Ahmad et al. 2015). To assess the role of hypoxia in causing the observed effects of Cl_2_ in this study we provided supplemental 40% oxygen to Cl_2_-exposed rats post-Cl_2_ exposure. Supplemental oxygen promptly reversed decreased oxygen saturations, however, the heart rates remained significantly decreased at 20 h postexposure (Fig.4A and B). On echocardiography, there was a return of EF, of the Cl_2_-exposed animals after oxygen supplementation, to those of controls coupled with (and probably explained by) an increase in LVESV toward those of controls (Fig.4C and D). However, indices of LV diastolic dysfunction such as *E*/*A* and *E*/*E*′ remained unchanged upon oxygen supplementation (Fig.4E and F).

We also measured ex vivo heart function with retrograde perfusion with Cl_2_ reactant taurochloramine (prepared as we described previously (Ahmad et al. 2015)) to assess the direct cardiotoxic effects of Cl_2_ reactants. We demonstrated occurrence of ∽1–2 *μ*mol/L chloramine in the plasma of Cl_2_-exposed rats after 30 min of exposure (Ahmad et al. 2015). However, higher concentrations of chloramine may occur, acutely or with higher concentration Cl_2_ exposures, along with other more potent reactants such chlorolipids. Perfusion with 100 *μ*mol/L chloramine resulted in >90% loss of force of contraction within 15 min (Fig.4G). Interestingly, even lower concentration (30 *μ*mol/L) of chloramine caused a gradual decline in the cardiac force of contraction and beats. Further lower concentration of chloramine (10 *μ*mol/L) though not significantly altering the force of contraction, was associated with a reduction in the frequency of beats during the observed 30 min duration.

## Discussion

Cl_2_ inhalation causes severe injury to the lungs along with pulmonary and systemic vasculature (Samal et al. 2010; Honavar et al. 2011, 2014). Inhalation of oxidant gases such as ozone, or free radicals potentially derived from combustion of hydrocarbons especially Cl_2_-containing hydrocarbons, compromise cardiac function (Pham et al. 2009; Lord et al. 2011; Rappold et al. 2011; Devlin et al. 2012). We demonstrated loss of cardiac ATP, SERCA activity, and apoptotic cardiomyocyte death within 30 min after Cl_2_ (500 ppm) inhalation in rats (Ahmad et al. 2015). We also demonstrated the existence of high concentrations of potentially stable by-products of the reaction of Cl_2_ with amines (chloramines) in the plasma of Cl_2_-exposed rats (Ahmad et al. 2015). These products are reactive and cause systemic injury; of particular interest is the demonstration of oxidation and chlorination of cardiac SERCA, which sequesters Ca^2+^ into the sarcoplasmic reticulum (SR) after its cytosolic release. Mishandling of Ca^2+^ due to SERCA dysfunction causes Ca^2+^ overload and serious cardiac injury in the form of cardiac remodeling, cardiac dilatation and failure (Boardman et al. 2014). Loss of ligand-induced Ca^2+^ mobilization and enhanced basal cytosolic Ca^2+^ levels (indicating Ca^2+^ overload) was observed in cardiomyocytes isolated from hearts of Cl_2_-inhaling rats and more recently in human airway smooth muscle cells exposed to Cl_2_ (Ahmad et al. 2015; Lazrak et al. 2015). Ca^2+^ overload can also activate proteases such as calpains that may exacerbate the injury by hydrolyzing the myocyte cytoskeletal intermediate filaments and other structural contractile proteins. Indeed we demonstrated increased troponin I levels in the circulation (Ahmad et al. 2015). In this study inhalation of Cl_2_ resulted in increased lactate in the coronary sinus of the rats. There was also an attenuation of myocardial contractile force in an ex vivo (Langendorff technique) retrograde perfused heart preparation. A reduction in systolic and diastolic blood pressure as well as a significant left ventricular systolic and diastolic dysfunction was observed. Higher concentration Cl_2_ exposure (600 ppm) was associated with biventricular failure and death. Cardiac mechanical dysfunction persisted despite increasing the inspired oxygen fraction concentration in Cl_2_-exposed rats to ameliorate hypoxia that occurs after Cl_2_ inhalation. Similarly ex vivo cardiac mechanical dysfunction was reproduced by exposure to chloramine (a potential circulating Cl_2_ reactant product).

Lactate has been shown to accumulate under conditions of myocardial ischemia and has been sought as a marker of lethal myocardial injury (Vogt et al. 2002). However, lactate may not always be due to anaerobic metabolism as increased metabolic rates under stress may elevate lactate under aerobic conditions (Garcia-Alvarez et al. 2014). Similarly, increased Na^+^-K^+^ ATPase activity may also cause increased lactate production in the absence of anaerobic conditions (Zhan et al. 1998). Increases in Na^+^-K^+^ ATPase accompany loss of SERCA activity as we have previously shown with Cl_2_ inhalation (Louch et al. 2010; Ahmad et al. 2015). A potential explanation may be cardiomyolisis (muscle tissue breakdown) of the heart with formation of creatine that further metabolizes to creatinine (Watson et al. 2011; Liu et al. 2013). Since we do not have cardiac creatinine extraction across the myocardium, elevated CS creatinine could also reflect a cardio-renal interaction. At present the implication of this finding is unknown and needs to be explored in future studies.

The decrease in the *E*/*A* and a significant reduction *E*/*E*′ could be due to decreased cardiac SERCA activity that we previously observed upon Cl_2_ inhalation (Ahmad et al. 2015). At the time of echocardiography (under anesthesia) the heart rate was not significantly different between groups (0 and 500 ppm) and hence we do not believe that it could contribute to the EA results. Moreover, a decline in heart rate, by reducing A wave velocity, would under-, rather then over-, estimate diastolic dysfunction. We speculate that the augmentation in LV shortening indices in vivo is due to the decrease in BP, which may in turn be due to a direct effect of Cl_2_ on vascular tone. Such hyperdynamic circulation as a consequence of impaired myocardial contracitility has been observed before (Laffi et al. 1997).

Taken together, our data indicate that a higher Cl_2_ exposure (600 ppm, 30 min) is associated with severe cardiac chamber dilatation and lethal myocardial depression. On the other hand, Cl_2_ exposure at 500 ppm resulted in a hyperdynamic LVEF in vivo despite intrinsic myocardial depression ex vivo. We posit that the unloading effect of a decrease in BP together with an expected increase in adrenergic drive over-rides the decrease in cardiac SERCA activity (previously demonstrated by us Ahmad et al. (2015)). However, the LV relaxation abnormality persisted and was not improved by reversal of hypoxia in vivo. Further studies are needed to elucidate the long-term effects of Cl_2_-induced cardiac dysfunction in humans and to investigate the role of different therapeutic targets in ameliorating this systolic and diastolic dysfunction. A therapeutic approach that alleviates both pulmonary and cardiac effects, rather than a sole pulmonary effect, may prove valuable in mitigating Cl_2_ inhalation-induced morbidity and mortality.
